# Consumer Perceptions of Meat Redness Were Strongly Influenced by Storage and Display Times

**DOI:** 10.3390/foods10030540

**Published:** 2021-03-05

**Authors:** Maddison T. Corlett, David W. Pethick, Khama R. Kelman, Robin H. Jacob, Graham E. Gardner

**Affiliations:** 1 Australian Cooperative Research Centre for Sheep Industry Innovation, Armidale 2350, Australia; D.Pethick@murdoch.edu.au (D.W.P.); K.Kelman@murdoch.edu.au (K.R.K.); G.Gardner@murdoch.edu.au (G.E.G.); 2 College of Science, Health, Engineering and Education, Murdoch University, Murdoch 6150, Australia; 3 Department of Primary Industries and Regional Development, South Perth 6151, Australia; robin.jacob@dpird.wa.gov.au

**Keywords:** fat, aging, breeding value, sheep, muscle, colour, growth, leanness, myoglobin

## Abstract

Lamb (*n* = 79) meat colour was scored by 879 untrained consumers using a scale of 0 (brown) to 100 (red). This consumer colour score (CCS) was obtained on *m. longissimus lumborum* (loin) and *m. semimembranosus* (topside), stored for short (5–7 days), medium (33–35 days), and long periods (110–112 days) and a retail display time of up to 4 days. Consumers perceived topside to be less red initially and changed from red to brown more rapidly when stored for the long-storage period (*p* < 0.01). Whereas, the initial CCS of loin samples were similar across the storage periods (*p* > 0.05). CCS and the instrument measure oxy/met (reflectance of light at wavelengths 630 nm and 580 nm) had a low correlation coefficient of 0.33 (*p* < 0.01). The propensity for lamb growth and leanness indicated by sire breeding values for lamb weight, eye muscle depth, eye muscle fat depth, and loin intramuscular fat had varied and inconsistent effects on CCS. Therefore, even the selection on CCS.

## 1. Introduction

The colour of meat on retail display has a critical influence on consumer purchasing decisions [1,2]. Consumers associate the colour of meat as an indicator of quality and freshness [1]. Consumers expect bright red meat to be fresher and of higher quality, whereas pale, discoloured, or darker meat is perceived by consumers to be nearing spoilage or of poorer quality [1]. Oxygen exposure causes the appealing bright red colour of meat by converting deoxymyoglobin into the red pigment known as oxymyoglobin. Continued exposure of the meat surface to oxygen causes oxidative metabolism and the subsequent generation of free radical by-products which then cause the oxidation of myoglobin into the brown pigment metmyoglobin [3]. 

Lamb meat in the Australian industry is typically stored chilled under vacuum packaging for a minimum of five days for domestic markets and thirty days or more for international markets due to shipping and transportation time. Two days is the typical retail display time of lamb meat when displayed in oxygen permeable polyvinyl chloride overwrap. After this time, retailers discount the meat to avoid rejection by consumers due to discolouration which represents a large economic loss to the lamb industry [4,5]. The *m. semimembranosus* is a more oxidative muscle that changes from red to brown quicker than the *m. longissimus lumborum* [6,7]. 

The rate at which the surface of meat changes from red to brown is the main factor limiting the retail display time and can be measured using the HunterLab. CIE-L * (black-white), a * (red-green) and b * (blue-yellow), and the ratio of reflectance of light at wavelengths 630 nm and 580 nm, also known as oxy/met, depicts the redness to brownness of a meat surface. In this study, the participants were also asked to score meat colour on a brown to red scale. 

Instruments can detect a noticeable colour difference due to retail display time [8,9], cut of meat [10], and storage period before display [11]. The impact that storage time has on retail display colour in lamb meat has been investigated previously [11], but only for storage periods up to 60 days. In this case, redness, indicated by oxy/met, decreased more rapidly in the *m. longissimus lumborum* and *m. semimembranosus* of 60-day aged lamb meat compared to 5-day aged meat [11]. However, storage periods longer than 60 days have not been investigated, yet given the demonstrated trends we would expect that the colour would continue to deteriorate. Crucially, this previous work quantified these effects using instrument measures reporting oxy/met and a *, but not using untrained consumers. Holman et al. [12] found that consumer acceptance of beef colour was best predicted from a * values however, this was a web-based study using photographs of beef *m. longissimus lumborum*. Given that these instruments measure colour changes detectable by the human eye [13], we expect that the data from untrained consumers would reflect similar results, highlighting that consumers perceive worse meat colour in lamb meat stored for long periods. 

Australian Sheep Breeding Values (ASBVs) allow lamb producers to rank sires genetically for growth, carcass composition, and eating quality traits. ASBVs used by the industry include post-weaning weight (PWT), post-weaning eye muscle depth (PEMD), post-weaning fat depth over the loin area (PFAT), and sire intramuscular fat percentage (IMF) in the loin. The PWT, PFAT, and PEMD ASBVs are used to select for rapid lean growth [14,15], while the sire IMF ASBV increases phenotypic IMF which enhances consumer eating quality [16,17]. These ASBVs have also been shown to change the colour of meat during retail display. Kelman et al. [18] observed selection for high PEMD reduced oxidative capacity of muscle, indicated by reduced isocitrate dehydrogenase (ICDH) activity and reduced myoglobin concentration [19], resulting in less red lamb meat. Contrary to this, Calnan et al. [20] found increasing PEMD had minimal effect on meat colour for the majority of lambs, yet reduced meat redness (a *) and chroma were still observed in one breed type. Calnan et al. [20] also showed no association between sire PWT and lamb loin colour. Selection for lower sire PFAT reduced ICDH activity and myoglobin concentration [18,19,21], and resulted in less type IIX glycolytic fibres [6], which would result in a less red appearance. This instrumental evidence suggests that it is likely that the corresponding consumer perception of colour will also be impacted, yet this evidence has not yet been reported in the literature.

In this study, we compared the effects of storage period and retail display time on colour measured by consumer colour scores (CCS) and oxy/met. This was measured in the loin, extracted from the *m. longissimus lumborum*, and topside, extracted from the *m. semimembranosus*, from lambs that were the progeny of sires with divergent ASBVs. We hypothesised that meat stored for longer periods would have lower CCS which will decline more rapidly during retail display. We also hypothesised that selection for growth (high sire PEMD and sire PWT) and leanness (low sire PFAT and sire IMF) would reduce CCS and that CCS would be higher in the loin than the topside. 

## 2. Materials and Methods

### 2.1. Experimental Design

The experimental design was a 2 × 3 × 4 factorial design, which consisted of two cuts of meat, three storage periods and four retail display times. The two cuts of meat were the loin and topside. The three storage periods were short (5 and 7 days), medium (33 and 35 days), or long (110 and 112 days), representing current domestic and international storage periods for Australian lamb meat. The four retail display times were 1, 2, 3, or 4 days under simulated retail overwrap display. The redness of meat samples under retail display was scored by 879 untrained consumers. Samples from the short and medium-storage periods were chilled similar to domestic refrigeration at 3–4 °C, while long-storage samples were chilled similar to extended export refrigeration temperature protocols at −1 °C.

Samples were collected from 79 lambs in the Meat and Livestock Australia’s Genetic Resource Flock based at Katanning Research Facility, Department of Primary Industry and Regional Development, Katanning, Western Australia. The lambs were the progeny of 41 Terminal (Poll Dorset, Suffolk, Texel, and White Suffolk) sires mated to Merino (*n* = 36) and Commercial Maternal (*n* = 41) dams. Sires were represented by a minimum of one progeny and a maximum of three progeny per sire. Lambs ranged from 279–303 days of age at slaughter, and 26 were females and 53 were castrated males. Lambs were maintained under extensive pasture grazing, with hay, grain or feedlot pellets supplemented when pasture supply was limited. Further information on genetic selection, lamb management, and feeding has been described previously [22,23]. The day prior to slaughter the lambs were held in yards for six hours, and then weighed and transported to a commercial abattoir. This is standard procedure at the abattoir, however, this live weight measurement was not included in the analysis. The lambs were then rested overnight in holding pens and slaughtered the following day.

### 2.2. Sample Collection and Carcass Measures

**Figure 1:**
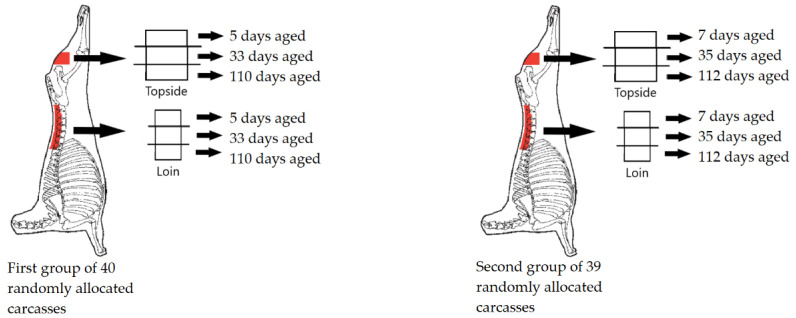
An example of loin and topside sample allocation to different storage periods for the two different groups of carcasses.

Lambs were slaughtered at a commercial abattoir using electrical head stunning followed by exsanguination. Thirty minutes after dressing all carcasses were electrically stimulated with a medium voltage system [24] and trimmed according to AUS-MEAT specifications [25]. Hot carcass weight was measured immediately after slaughter, averaging 20.11 kg (Standard deviation = 1.74) and GR tissue depth (11 cm from the midline to the lateral surface of the 12th rib) averaging 6.68 mm (Standard deviation = 2.01). Eye muscle area was measured between the 12th and 13th rib. Carcasses were chilled overnight at 3–4 °C, and 24 h post-mortem, the *m. longissimus lumborum* and *m. semimembranosus* was removed from each carcass. The *m. longissimus lumborum* was excised between the 12th/13th rib and the caudal end of the *m. longissimus*, and overlaying subcutaneous fat was removed (Loin; AUSMEAT 5150). The loin was weighed separately to the subcutaneous fat layer covering the muscle (loin muscle weight and loin fat weight). About 40 g of loin muscle from the caudal (lumbar-sacral) portion of the muscle was collected from each carcass and stored at −20 °C until freeze-drying with a Cuddon FD 1015 (Cuddon Freeze Dry, NZ). The concentration of IMF in the loin was then determined using a near-infrared procedure in a Technicon Infralyser 450 (19 wavelengths) using the method described by Perry et al. [26] and expressed as percentage fat. The *m. semimembranosus*, cap off, was also excised from the carcass (Topside; AUSMEAT 5077). This meant the *m. gracilis* and *m. adductor* was removed in the abattoir so that consumers were scoring the *m. semimembranosus*. Each loin and topside were sliced into three samples (50 mm in length, 50 mm in width, and 30 mm in depth) and individually vacuum packed. Samples were then aged in vacuum packaging for one of three storage periods; short (5 and 7 days), medium (33 and 35 days), or long (110 and 112 days). Lamb meat can be stored for up to 12 weeks, vacuum packaged and chilled at low temperatures (−1 °C–0 °C) prior to retail display [27]. The long storage period adopted in this study represents this extended storage period of lamb meat which facilitates the transportation of chilled meat to distant overseas markets. Loin and topside samples from one animal would be allocated to the preceding days (5, 33 and 110). While loin and topside samples from another animal would be allocated to the later days (7, 35 and 112), demonstrated in Figure 1. This staggering within a storage period, such as 5 and 7 days storage prior to display, enabled consumers to visually compare samples with varying redness due to a range in time spent under retail display. 

After the allocated storage period, loin samples were butterflied (to 15 mm thickness) and topside samples were re-sliced (to approximately 30 mm depth) perpendicular to muscle fibre orientation to expose a freshly cut surface. Each sample was placed on a black Styrofoam tray (12 cm × 12 cm) with the freshly cut surface facing upwards before being overwrapped with oxygen-permeable polyvinyl chloride film (Resinite “DHW” Meat AEP, 15 µm thickness, oxygen transmission rate of 35,650–46,500 cc/m^2^/24 h). Samples were then placed under simulated retail display for four days on a flat horizontal surface within a walk-in chiller fitted with 8 Nelson Fluorescent Meat Display BRB Tubes, 58 W and 1520 mm in length, suspended 1.5 m above the samples. The light intensity provided was 1000 Lux as measured by a Digitech Lux Light Meter QM1587. The temperature within the walk-in chiller was set at 4 °C with no defrost cycle and fluctuated between 2 and 6 °C. These light and temperature conditions were designed to simulate those commonly encountered in Australian retail stores. The position of samples under display were rotated daily to limit position effect.

**Figure 2:**
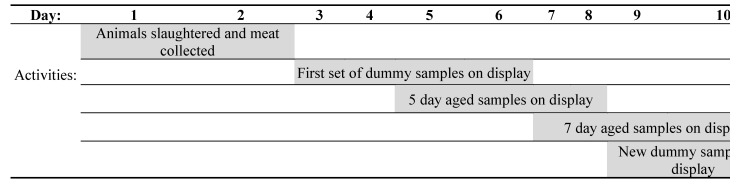
Demonstration of the experimental design for the number of days aged, retail display time, and inclusion of dummy samples within the short storage period.

To provide a wider range of redness, additional lamb meat samples purchased from a commercial butcher were included. These “dummy” samples were subjected to a retail display time of 1–3 days greater than experimental samples (Figure 2). For example, dummy samples were placed under display on day 3 of the experiment and on day 4 and were less red than the experiment samples that placed on display on day 5 when viewed by consumers. Dummy samples were also for comparison with the later aged samples (Figure 2). Later aged samples, such as day 7 short storage samples, were under display for 3 or 4 days when the dummy samples were freshly sliced and placed under display with the experimental samples allowing consumers to view a range of redness in the samples at any given time. This is consistent with the method of Khliji et al. [13] to create a range in meat colour from brown to red for consumers to visually assess.

### 2.3. Sire Breeding Values

Sire breeding values are derived from measures on the individual animals, the performance of their relatives, and appropriate genetic parameters [28]. Sire PWT values were based on lamb live weights corrected to 225 days of age [20]. Sire PFAT measures were calculated from ultrasound measurements of fat depth at the c-site (45 mm from the mid-line over the 12th rib) and adjusted to 60 kg live weight [28]. Sire PEMD values were calculated from ultrasound measurements of the loin muscle depth at the level of the 12th rib and adjusted for live weight [29]. 

Sire IMF values were estimated by Sheep Genetics using all available pedigree, genomic, and phenotypic information available for the sires and their relatives. Phenotypic IMF observations are the primary driver for the sire IMF breeding value and were measured in their progeny by taking samples from the *m. longissimus lumborum* between the 12th and 13th rib and measuring IMF (described previously). Some additional carcass traits and live animal measures such as live weight and ultrasound scanning also contribute via genetic correlations.

### 2.4. Apprasel of Meat Colour

Consumers were untrained and different individuals were recruited for each viewing day and storage period. The staggering of samples within a storage period meant there were six consecutive viewing days for each storage period (Figure 2). Each sample was viewed and scored by 10 consumers each day, for four days. Each consumer assessed 24 randomly allocated meat samples on the specified day with a CCS of each sample from 0 (brown) to 100 (red) by placing a mark on a line 10 cm long. 

In addition to scoring meat colour, consumers completed a demographic questionnaire at the time of meat appraisal. Recruitment required consumers to be aged between 18–70 years old and not colour blind. The questions asked were:

1. Age group: 18–19 years, 20–25 years, 26–30 years, 31–39 years, 40–60 years, 61–70 years;
2. Gender: male, female;
3. Primary purchaser for the household: yes, no;
4. Consumption frequency of red meat (including sheep meat, beef, and pork): daily, 4–5 times a week, 2–3 times a week, weekly, monthly, never.

### 2.5. Oxymyoglobin to Metmyoglobin Ratio

Light reflectance was measured at the meat surface using a HunterLab Mini Scan EZ (model No. 45/0-L, Hunter Associates Laboratory Inc., Reston, VA, USA) with an aperture size of 25 mm. The instrument was calibrated with black and white ceramic tiles according to the manufacturer’s directions. The light source was set to “D65” and the observer set to 10°. The oxy/met value can be calculated from the reflectance readings at 580 and 630 nm [30]. 580 is the reflectance minimum for red oxymyoglobin while 630 nm is the maximum reflection of pure oxymyoglobin. An oxy/met value greater than 4 suggests that the majority of the pigment is the red oxymyoglobin, while a value approaching 1 suggests mostly brown metmyoglobin. Three replicate measures were taken of the meat surface with the instrument head rotated 90° for each measurement. The three replicate measures for each sample were averaged for analysis. The operator also made the effort to avoid areas of dense subcutaneous fat or connective tissue during measurements.

### 2.6. Statistical Analysis

CCS data was analysed using linear mixed effects models (SAS Version 9.1, SAS Institute, Cary, NC, USA). The base model included fixed effects for storage period (short, medium, long), retail display time (1, 2, 3, 4 days), and cut (loin, topside). Meat identification number within animal identification by sire identification and consumer identification was included as random terms in the model. All relevant first-order interactions between fixed effects were tested and non-significant interactions (*p* > 0.05) were removed in a stepwise manner. This analytical approach was then applied to oxy/met.

Phenotypic covariates including hot carcass weight, GR tissue depth, and IMF were then tested individually in the model along with all first-order interactions to assess their association with CCS. Loin fat weight, loin weight, and eye muscle area were tested using this same approach, although in this case hot carcass weight was also included. This enabled these terms to act as “proxies” for whole carcass fatness and muscularity. The phenotypic covariates described above were then included individually in the ASBV model to establish whether they accounted for any of the ASBV effects. 

The association between CCS and sire ASBVs for PFAT, PEMD, PWT, and sire IMF were also tested individually as covariates. The sire ASBVs for PFAT, PEMD, and PWT were tested together in the base model along with all relevant first order interactions with fixed effects. The effect of sire IMF on CCS was tested separately from the base model. Phenotypic IMF was then added to see if the effect of sire IMF on CCS changed. 

The association between CCS and oxy/met was tested for a simple and partial correlation using multivariate regression analysis (SAS Version 9.1). In this multivariate regression, CCS and oxy/met were fitted as dependent variables, and the cut of meat (loin, topside) was fitted as a fixed effect.

## 3. Results

The mean, standard deviation, and range for the carcass measurements and sire ASBVs are shown in Table 1. 

### 3.1. The Effect of Cut, Storage Period, and Retail Display Time on Consumer Colour Score

**Figure 3:**
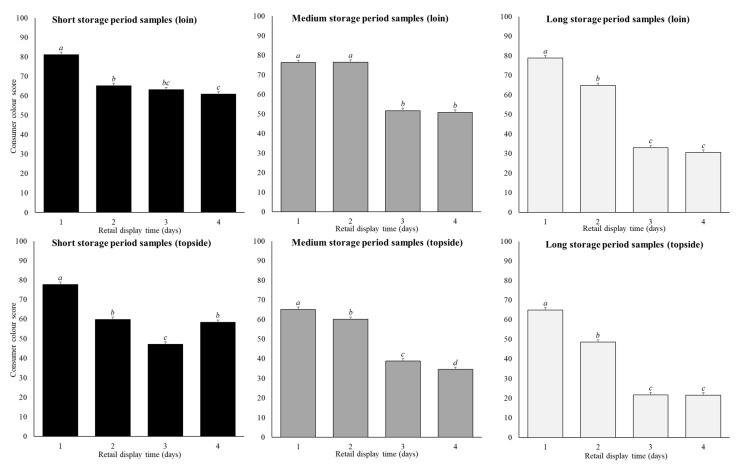
Predicted means (±SE) for the storage period (short, medium, long), and retail display time (1, 2, 3, 4 days) for loin and topside samples on consumer colour scores in the base model. Within a cut and storage period the annotations (**a**–**d**) that differ indicate significant difference (*p* < 0.05) across retail display.

Cut, storage period and retail display time affected CCS (*p* < 0.01) (Table 2; Figure 3). On average across all retail display and storage periods, the loin samples (61 ± 0.7 units) scored higher (*p* < 0.01) compared to the topside samples (50 ± 0.7 units). During retail display, CCS across both cuts and all storage periods reduced (*p* < 0.05) by an average of 12 units between day 1 (74 ± 0.7 units) and day 2 (62 ± 0.7 units). On day 3, CCS reduced (*p* < 0.05) by an average of 19 units (43 ± 0.7 units) followed by minimal difference (*p* > 0.05) between days 3 and 4. 

The reduction in CCS across the four-day retail display was greater in samples stored for longer storage periods (*p* < 0.01; Figure 3). For the short-storage loin samples, CCS decreased (*p* < 0.01) by an average of 20 units between day 1 to day 4 of retail display. On the first day of retail display, the medium and long-storage loin cuts showed similar CCS values to the short-storage cuts, but then fell by 25 units and 48 units across the four days of retail display (Figure 3). Likewise, for the short-storage topside cuts, the CCS decreased (*p* < 0.01) by 19 units between day 1 to day 4 of retail display, while for the medium and-long storage topside cuts decreased (*p* < 0.01) by 31 and 43 units (Figure 3). In this case, the CCS was not the same on day 1 of retail display, with short storage topside CCS values on average being 13 units higher than the medium and long-storage topside samples. Overall, the decrease in CCS across the retail display period was always greater in the loin compared to the topside (Figure 3). This meant that in short-storage loin and topside product, samples displayed for up to 4 days generally scored above 50 on the scale of 0 to 100, thus were perceived more red than brown. Whereas in the medium and long-storage products, samples displayed for 1–2 days scored above 50, with those displayed for 3–4 days generally scoring at or below 50, and therefore perceived as brown.

### 3.2. Oxymyoglobin to Metmyoglobin Ratio

**Figure 4:**
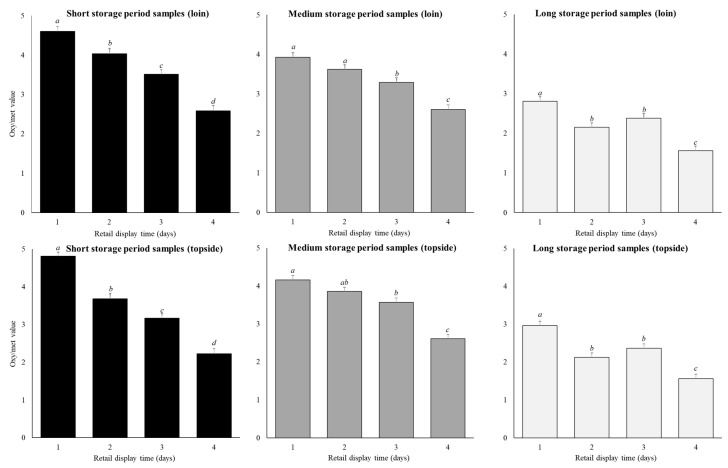
Predicted means (±SE) for the storage period (short, medium, long), and retail display time (1, 2, 3, 4 days) for loin and topside samples on oxy/met values. Within a cut and storage period the annotations (**a**–**d**) ) that differ indicate significant difference (*p* < 0.05) across retail display.

Retail display time affected oxy/met values (*p* < 0.01), showing similar trends to the CCS (Table 2; Figure 4). Across both cuts and all storage periods, oxy/met continuously reduced (*p* < 0.05) by as much as 1.7 units from day 1 to day 4 (Figure 4). In contrast to the CCS data, the loin (3.1 ± 0.1 units) and topside samples (3.1 ± 0.1 units; Figure 4) on average had similar (*p* > 0.05) oxy/met.

The reduction in oxy/met across the four-day retail display was greater in samples stored for shorter periods (*p* < 0.01; Figure 4), a trend opposite to that seen for CCS. Thus, for the short-storage loin cuts, oxy/met decreased (*p* < 0.01) by 2.0 units between day 1 to day 4 of retail display, while for the long-storage loin cuts this decrease (*p* < 0.01) was only 1.2 units. Similarly, for the short-storage topside cuts, oxy/met decreased (*p* < 0.01) by 2.6 units between day 1 to day 4 of retail display, while for the long-storage topside cuts this decrease (*p* < 0.01) was only 1.4 units. In general, the decrease in oxy/met across retail display was always greater in the topside compared to the loin, again opposite to the trend seen with CCS. 

The simple correlation of CCS and oxy/met was 0.33 (*p* < 0.01) and the partial correlation was 0.34 (*p* < 0.01). 

### 3.3. Association of Carcass Measurements with Consumer Colour Score

Inclusion of loin fat weight (with hot carcass weight also in the statistical model) did not substantially change the base model but did influence the CCS in some cuts at different storage periods albeit inconsistently. Increasing loin fat weight decreased CCS by 0.07 (±0.02) units per gram of loin fat weight, but only in the long-storage loin samples (*p* < 0.05). As loin fat weight increased over the 150 g range, this was equivalent to a reduction of 10.4 units in CCS. Other terms in the base model remained unchanged with the inclusion of loin fat weight.

The other carcass measures tested, including phenotypic intramuscular fat, hot carcass weight, GR tissue depth, loin weight, and loin eye muscle area demonstrated no association with CCS.

### 3.4. Association of Sire ASBVs on Consumer Colour Score

Sire ASBVs influenced CCS, however, these effects were inconsistent and varied across cuts and storage periods. The effect of sire PFAT was evident in the medium-storage loin and topside samples with CCS reducing by 5.5 and increasing 5.5 units across the range of PFAT (*p* < 0.05). The effect of sire PWT was only evident in the medium-storage loin reducing CCS by 8.5 units over the PWT range (*p* < 0.05). The effect of sire PEMD was only evident in the long-storage topside with CCS reducing by 7.3 units over the PEMD range (*p* < 0.05). When sire PWT, PEMD, and PFAT were included individually in the base model, in general CCS remained unchanged in comparison to the combined ASBV model. The only exception was that sire PEMD increased CCS in the short-storage loin. When phenotypic carcass measurements were included in these models, the ASBV effects remained unchanged.

When the sire IMF breeding value was included individually in the base model its association (*p* < 0.01) with CCS varied between the different cuts and storage periods. The effect of sire IMF breeding value was evident in the medium and long-storage topside samples with CCS increasing by 3.7 (±1.3) and 2.7 (±1.3) units per % of sire IMF (*p* < 0.01). Over the 2.23% range in sire IMF, this was equivalent to an increase of 7.1 and 5.3 units in CCS. When the phenotypic IMF was added to this model the association between CCS and sire IMF remained unchanged. 

### 3.5. Consumer Demographics

The majority of the consumers were aged between 20–25 years old, identified as female, and were the primary purchaser for the household (Table 3). The highest proportion of consumers said they consume red meat 2–3 times a week, followed by those stating they consume red meat 4–5 times a week.

## 4. Discussion

### 4.1. Effect of Storage Time on Consumer Colour Score

As hypothesised, topside samples stored for longer periods had lower CCS on day 1 of retail display. Yet contrary to this, the loin samples did not differ on day 1. Generally, the *m. semimembranosus*, from which the topside is derived, has a higher proportion of oxidative type I muscle fibres which express higher levels of oxidative enzymes, such as ICDH, compared to loin samples [6]. This higher proportion of oxidative muscle fibres found in the topside increases oxygen metabolism post-mortem, resulting in increased metmyoglobin formation [31], lipid oxidation [32], and reduces meat redness [4]. This supports our finding that initial topside CCS was lower following longer storage periods, whereas initial loin CCS remained consistently high regardless of storage time. The constantly high initial CCS of loin samples is likely due to mitochondrial activity declining within the meat sample with increased storage time, resulting in deeper bloom depth. This theory was originally proposed by MacDougall and Allen [33], where aged meat had redder initial meat colour due to lower mitochondrial oxygen consumption rates which enabled deeper oxygen penetration into the meat. Likewise, Tang et al. [34] showed beef hearts at 60 days post-mortem had mitochondria that still consumed oxygen, but at much lower rates than mitochondria isolated at 4 days post-mortem. This highlights that loin samples, when previously stored for up to 112 days, can still be on display for sale for two days before CCS rapidly declines and the product begins to discolour. 

Alternatively, and consistent with our hypothesis, both loin and topside CCS declined more in meat stored for longer periods during retail display. This rapid decline in meat redness following a prolonged storage period was likely due to a higher rate of lipid oxidation and free-radical presence during retail display [11,32]. A review by MacDougall and Allen [33] found aged beef meat has brighter colour immediately after blooming, because of increased light scatter and deeper oxygen penetration, but redness rapidly declines during retail display. Likewise, Ponnampalam et al. [11] found aging lamb loin samples for 60 days reduced redness (a * and oxy/met) during the four days of retail display. Ponnampalam et al. [35] showed samples aged for four weeks were redder (higher a * and oxy/met) than fresh samples during the retail display. However, the fresh samples used by Ponnampalam et al. [35] did not follow the Meat Standards Australia guidelines [36] which stipulate all lamb must be pre-aged in vacuum for a minimum of 5 days post-slaughter, before being placed on retail display. Nevertheless, an extended storage period has implications for chilled meat products exported from Australia via sea freight. Across all storage periods and cuts, on average sample CCS decreased during retail display. This finding was consistent with previous literature where lamb meat colour was instrumentally measured [8,10,11,13]. In the current study, samples receiving a CCS above 50 consumers perceived as more red than brown, and thus potentially more desirable for purchasing. Therefore, consumers could purchase loin products from day 1 up to day 4 of retail display, after having been stored for five days, as the loin samples generally scored above 50. This goes beyond the standard practice of discounting meat after two days of retail display which is currently used by retailers. This extended selling-time would increase retailer profits as the economic losses from prematurely discounting meat would be reduced. 

### 4.2. Effect of Cut on Consumer Colour Score

As hypothesised, CCS was higher in the loin than the topside across all storage periods and retail display. Differences between loin and topside samples were likely due to variation in muscle fibre type and the associated changes in oxidative capacity, with these differences affecting meat colour and thus consumer perception. As previously noted, the topside has higher proportions of oxidative type I muscle fibres which increases oxygen metabolism post-mortem. The higher oxygen consumption rate increases metmyoglobin formation [31] and reduces meat redness [4]. This difference in meat colour between lamb loin and topside samples was shown previously using instrumental measures [10,37], thus it was expected that consumers would score loin samples higher than topside samples. This finding highlights the potential for retailers to set varying display times for different meat cuts based on their perceived redness, thus maximising the opportunity to sell them.

### 4.3. Oxymyoglobin to Metmyoglobin Ratio

The trends evident within the CCS results were typically reflected by oxy/met, for instance, oxy/met generally declined across retail display in both cuts. However, there were discrepancies between CCS and oxy/met within the findings of this study. The CCS values of long-storage loin samples on day 1 and 2 of retail display were relatively high in redness but then declined rapidly on day 3 and 4. In comparison, oxy/met showed that long-storage loin samples had values that were initially much lower than the short and medium-storage samples which also declined less during retail display. This suggests oxy/met does not capture what consumers perceived. Another discrepancy between CCS and oxy/met was for the comparison of cut. In this case, the loin CCS declined more across the retail display compared to the topside, yet for the oxy/met the opposite was seen. These discrepancies are supported by the relatively low simple and partial correlation coefficients between CCS and oxy/met, demonstrating that this association is far from perfect. Shorthose et al. [38] also reported a low correlation (*r* = 0.32) for beef meat. This highlights that consumers perceive more than just brown to red when assessing the colour of meat. It also highlights that consumer data, which is inherently variable, is difficult for objective instruments to accurately predict. This has been identified previously as Hopkins [39] found low correlations between consumer acceptability scores and L *, a * and b * values of lamb meat. Likewise, Morrissey et al. [40] found a poor relationship between consumer scores and oxy/met for lamb loin samples. These previous studies confirm low correlation between CCS and oxy/met, found in the current study, demonstrates that instrumental measures are unreliable proxies for consumer perceptions of meat redness. 

### 4.4. Carcass Measurements

As loin fat weight increased consumers perceived a decrease in redness, yet only in products from the long-storage period. One theory could be that higher fat levels are normally associated with higher phenotypic IMF levels [41], which in turn are associated with higher oxidative capacity [42]. This would result in increased metmyoglobin formation and reduced CCS. Yet, contrary to this we found that the direct measure of IMF within the loin was not associated with CCS. Alternatively, increased carcass fatness is an indicator of greater maturity, which in turn has been shown to increase muscle oxidative capacity [6,43], possibly resulting in a decreased CCS. 

There was no association between the other carcass measures and CCS. This may be attributed to the other carcass measures having no association with muscle oxidative capacity, and thus were not associated with CCS. These carcass measures included intramuscular fat, hot carcass weight, GR tissue depth, loin weight, and loin eye muscle area. This finding implies that carcasses with a large range of phenotypic attributes will not affect the redness of meat consumers perceived. Instead, it is the effects of storage and retail display time that have the most influence on CCS.

### 4.5. Sire ASBVs

When sire PFAT, PEMD, and PWT were included simultaneously in the base model, there was an association between sire PWT by storage period and cut on CCS. In partial support of our hypothesis, increasing sire PWT estimates were associated with reduced CCS, however, this effect was only evident in the medium-storage loin samples. This association was expected as increased sire PWT estimates have been linked to progeny reaching target slaughter weights faster, when less mature [44]. This reduced maturity is reflected in the reduced proportion of oxidative muscle fibre types and the subsequent oxidative capacity of the muscle [6,45] which may influence meat colour. In contrast, Kelman et al. [18] found increasing sire PWT estimates increased lamb loin ICDH activity, myoglobin concentration, and muscle oxidative capacity. Kelman et al. [18] speculated that the impact of sire PWT on muscle oxidative capacity could be limited to the saddle region as including loin muscle weight nullified the impact of sire PWT on ICDH activity and myoglobin concentrations in the study. The current study tested a range of carcass measurements, including loin muscle weight, and the association between sire PWT and CCS remained unchanged with the inclusion of loin muscle weight to the combined ASBV model. Interestingly, Calnan et al. [20] reported no association between sire PWT estimates and instrument colour measures of loin meat over retail display. This may be because Calnan et al. [20] only observed loin samples aged for five days prior to display, whereas the current study used both loin and topside cuts, and extended storage periods.

Increasing sire PEMD estimates were associated with reduced CCS in the long-storage topside samples, partially supporting our hypothesis. Kelman et al. [18] demonstrated similar findings as a selection for high PEMD, reduced muscle oxidative capacity, indicated by reduced isocitrate dehydrogenase (ICDH) activity and reduced myoglobin concentrations [18,19] which would result in a less red meat appearance. This association between sire PEMD and oxidative capacity was likely due to a shift in muscle fibre type with increased sire PEMD, increasing the proportion of type IIX glycolytic fibres [6,46], which would result in a less red meat appearance. In contrast, Calnan et al. [20] found increased sire PEMD was associated with varied and inconsistent effects on instrumental measures of redness (a * and chroma) between lambs of different sexes and breed types. 

Reducing sire PFAT estimates were associated with increased CCS for the medium-storage loin samples, yet reduced CCS for medium-storage topside samples. These results partially support our hypothesis as selecting for reduced PFAT reduced CCS; however, this was only present in medium-storage topside samples. Selecting for reduced sire PFAT has been previously associated with an increased proportion of glycolytic type IIX fibres [6] which are described as the white (fast glycolytic) muscle fibres. Likewise, reduced PFAT has been associated with reduced ICDH activity and myoglobin concentrations in the meat [18,19], and thus reducing oxidative capacity [42] which would cause the meat to appear less red during retail display. A similar finding was shown by Calnan et al. [20], where reduced sire PFAT estimates reduced meat redness (a *) in the terminal sired lambs. 

In partial support of our hypothesis, reduced sire IMF estimates reduced CCS, however, this association was only present in medium and long-storage topside samples. As sire IMF is a relatively new ASBV no published literature has tested its effect on meat colour. However, Calnan et al. [20] observed a similar association between phenotypic IMF and instrument measured colour, where an increase in IMF increased lamb loin lightness (L *), redness (a *), yellowness (b *), hue, and chroma. However, in the current study, the phenotypic IMF had no association with CCS. These results remain encouraging for the lamb meat industry as it indicates increased IMF will not harm the perceived redness of lamb meat on display by untrained consumers, yet will improve eating quality [16,17]. 

The associations between sire PWT, PEMD and PFAT estimates and CCS were independent of carcass measurements as the magnitude of these associations remained unchanged with the inclusion of carcass measurements in the model. The exception being the inclusion of hot carcass weight, loin eye muscle area, loin weight, and loin fat within the combined ASBVs model, which resulted in sire PWT affecting CCS in the long-storage topside samples which were previously non-significant. Likewise, the inclusion of phenotypic IMF into the combined ASBV model resulted in the association between CCS becoming non-significant in medium-storage topside samples, although the effect on CCS was minimal. Nonetheless, the sheep breeding values do not appear to be delivering their impact through their related phenotypes. This was supported by the fact that the inclusion of carcass measurements does not account for any further variation in CCS that is already explained by the combined ASBVs model. 

A positive association between sire PFAT and phenotypic IMF has been previously demonstrated, where reducing sire PFAT reduced IMF in the progeny [41]. However, the inclusion of phenotypic IMF in the combined ASBV model did not change the association between sire PFAT and CCS, suggesting that the impact of sire PFAT was not delivered through its association with phenotypic IMF. Similar findings were reported by Kelman et al. [18] where the inclusion of phenotypic IMF into the ASBV model did not diminish the positive association between sire PFAT on ICDH activity and myoglobin concentration, both indicators of oxidative capacity, and thus meat redness. Therefore, sire PFAT explains more of the variation in CCS than phenotypic IMF.

### 4.6. Industry Relevance

The storage periods tested in this study enable extrapolation of these results to several key domestic and international market scenarios. The short-storage samples where meat was aged for five days is relevant to domestic supply chains, with Meat Standards Australia guidelines [36] stipulating that all lamb must be aged for a minimum of five days before display. Alternatively, the medium and long-storage scenarios where meat was aged for 33–35 and 110–112 days are highly relevant to international markets. Countries such as Australia and New Zealand export chilled meat overseas to distant countries via sea freight, which will often extend to between 30 to 110 days before the meat is sliced and displayed at retail [47]. On this basis, it is crucial to understand the impact of extended storage on consumer perceptions of sheep meat as this represents a key international export for countries such as Australia. 

The four retail display times used in this study go beyond the standard two-day display period, enabling us to understand the association between consumer perception and instrument measures across greater extremes of colour and storage periods. A small number of lamb meat colour studies have assessed meat colour over a similar duration of display, but typically after a short storage period of about five days. Previously this was studied in the *m. longissimus*, and generally with no consumer data included [8,10,11,13]. Only Khliji et al. [13] recorded untrained consumer data but were limited to 541 consumers. In the Ponnampalam et al. [11] study, meat samples were stored up to 60 days, but no consumer data was recorded. In contrast, the current study examined the effect of prolonged storage periods in an additional cut and included data from a larger number of consumers. This will provide an excellent point of reference going forward for the red meat industry as many instrument colour studies exist, yet there is little untrained consumer data.

## 5. Conclusions

This study confirms meat stored for longer periods was initially perceived by consumers as less red, and then also declined more rapidly during retail display. However, the initial CCS of loin samples did not differ across storage periods. The correlation between CCS and oxy/met was low, indicating instrumental measures as unreliable proxies for consumer perceptions due to the variable nature of consumer data. Based on the findings from the current study, the practice of discounting lamb meat after 48 hours of retail display is justified to ensure acceptably red meat in the domestic market. However, the CCS of loin samples, aged for five days, were generally over 50 which suggests consumers still view them as more red than brown, and potentially likely to still purchase. This extended selling time could benefit retailers that currently lose profits from pre-emptively discounting after just two-days of retail display. 

Sire ASBVs were associated with CCS of lamb meat under retail display. However, the association was inconsistent, varied across cuts and storage periods, and was at most 8.5 units across the entire ASBV range. This means the selection of sires with diverse breeding values for growth and leanness will generally have no detrimental effect on consumer perceptions of meat redness. Overall, this is a reassuring finding for the Australian lamb meat industry as there are minimal impacts on consumer perceptions of meat colour when producers select for traits such as leanness and growth using ASBVs. 

## Figures and Tables

**Table 1:** Descriptive statistics of carcass measurements and sire Australian Sheep Breeding Values.

Variable	Mean	Standard Deviation	Range
**Carcass measurements**			
Intramuscular fat (%)	3.65	0.738	2.45–5.97
Hot carcass weight (kg)	20.11	1.742	16.70–25.50
GR tissue depth (mm)	6.68	2.011	3.00–11.00
Loin fat weight (g)	128.56	37.575	55.00–224.00
Loin weight (g)	308.37	49.078	223.00–491.00
Loin eye muscle area (cm^2^)	15.15	2.401	9.74–21.89
**Sire Australian Sheep Breeding Values**			
Post-weaning weight (kg)	13.42	2.191	8.94–17.91
Post-weaning eye muscle depth (mm)	2.09	1.242	−0.36–5.21
Post-weaning fat depth (mm)	−0.53	0.703	−2.00–1.06
Sire intramuscular fat (%)	−0.61	0.483	−2.01–0.22

**Table 2:** F-values for the effects of the storage period, cut, and retail display time on consumer colour scores and oxy/met.

Effect	NDF	Consumer Colour Scores F-Value	Oxy/Met F-Value
Storage period	2	224.97 *	95.34 *
Cut	1	175.52 *	ns
Retail display time	3	1748.44 *	511.17 *
Storage period * Cut	2	75.65 *	59.13 *
Cut * Retail display time	3	16.86 *	21.77 *
Storage period * Retail display time	6	150.8 *	43.21 *
Storage period * Cut * Retail display time	6	26.91 *	8.86 *

*: *p* < 0.01; ns: not significant; NDF: numerator degrees of freedom. There were 18,000 denominator degrees of freedom for all fixed effects and interactions for consumer colour scores and 16,000 for oxy/met.

**Table 3:** Percentage (and number) of consumers within each demographic and meat consumption category.

**Age**	**Percentage (and Number) of Consumers**
18–19 years	21.9 (192)
20–25 years	41.0 (360)
26–30 years	11.3 (99)
31–39 years	8.0 (70)
40–60 years	12.6 (111)
61–70 years	5.2 (46)
**Gender**	
Female	59.5 (522)
Male	40.5 (356)
**Primary purchaser for household**	
Yes	61.5 (540)
No	38.5 (338)
**Red meat consumption frequency**	
Daily	11.2 (98)
4–5 times a week	28.0 (246)
2–3 times a week	36.3 (319)
Weekly	15.0 (132)
Monthly	6.8 (60)
Never	2.6 (23)

## Abbreviations

ASBV	Australian Sheep Breeding Values
CCS	consumer colour scores
ICDH	isocitrate dehydrogenase
IMF	intramuscular fat percentage
Oxy/met	reflectance of light at wavelengths 630 nm and 580 nm
PEMD	post-weaning eye muscle depth
PFAT	post-weaning fat depth over the loin area
PWT	post-weaning weight
